# Polar tagging in the synthesis of monodisperse oligo(*p*-phenyleneethynylene)s and an update on the synthesis of oligoPPEs

**DOI:** 10.3762/bjoc.6.57

**Published:** 2010-06-01

**Authors:** Dhananjaya Sahoo, Susanne Thiele, Miriam Schulte, Navid Ramezanian, Adelheid Godt

**Affiliations:** 1 Bielefeld University, Faculty of Chemistry, Universitätsstr. 25, D-33615 Bielefeld, Germany

**Keywords:** alkyne protecting group, carbometalation, C–C coupling, phenyleneethynylene, polar tagging

## Abstract

One important access to monodisperse (functionalized) oligoPPEs is based on the orthogonality of the alkyne protecting groups triisopropylsilyl and hydroxymethyl (HOM) and on the polar tagging with the hydroxymethyl moiety for an easy chromatographic separation of the products. This paper provides an update of this synthetic route. For the deprotection of HOM protected alkynes, γ-MnO_2_ proved to be better than (highly) activated MnO_2_. The use of HOM as an alkyne protecting group is accompanied by carbometalation as a side reaction in the alkynyl–aryl coupling. The extent of carbometalation can be distinctly reduced through substitution of HOM for 1-hydroxyethyl. The strategy of polar tagging is extended by embedding ether linkages within the solubilising side chains. With building blocks such as 1,4-diiodo-2,5-bis(6-methoxyhexyl) less steps are needed to assemble oligoPPEs with functional end groups and the isolation of pure compounds becomes simple. For the preparation of 1,4-dialkyl-2,5-diiodobenzene a better procedure is presented together with the finding that 1,4-dialkyl-2,3-diiodobenzene, a constitutional isomer of 1,4-dialkyl-2,5-diiodobenzene, is one of the byproducts.

## Introduction

**Scheme 1:**
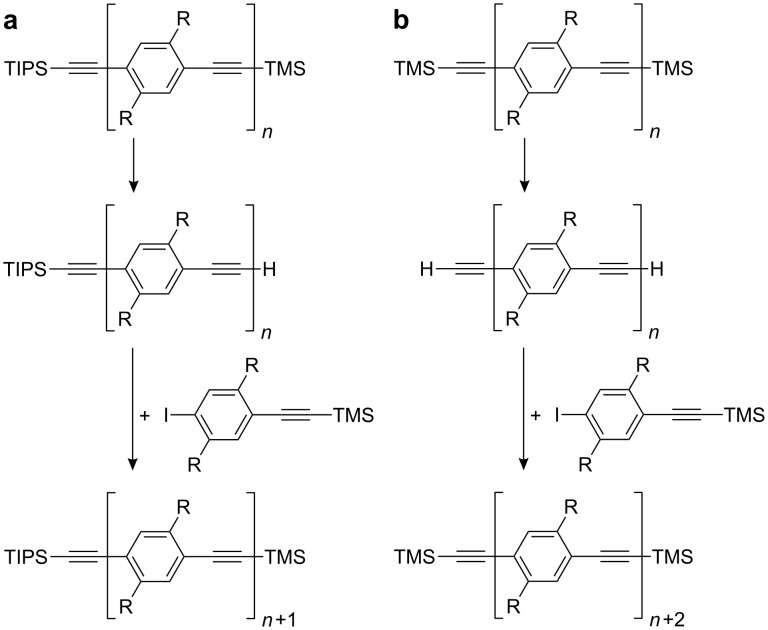
Synthesis of oligoPPEs by a unidirectional (a) or bidirectional (b) repeating unit by repeating unit approach. R denotes solubilising substituents.

**Scheme 2:**
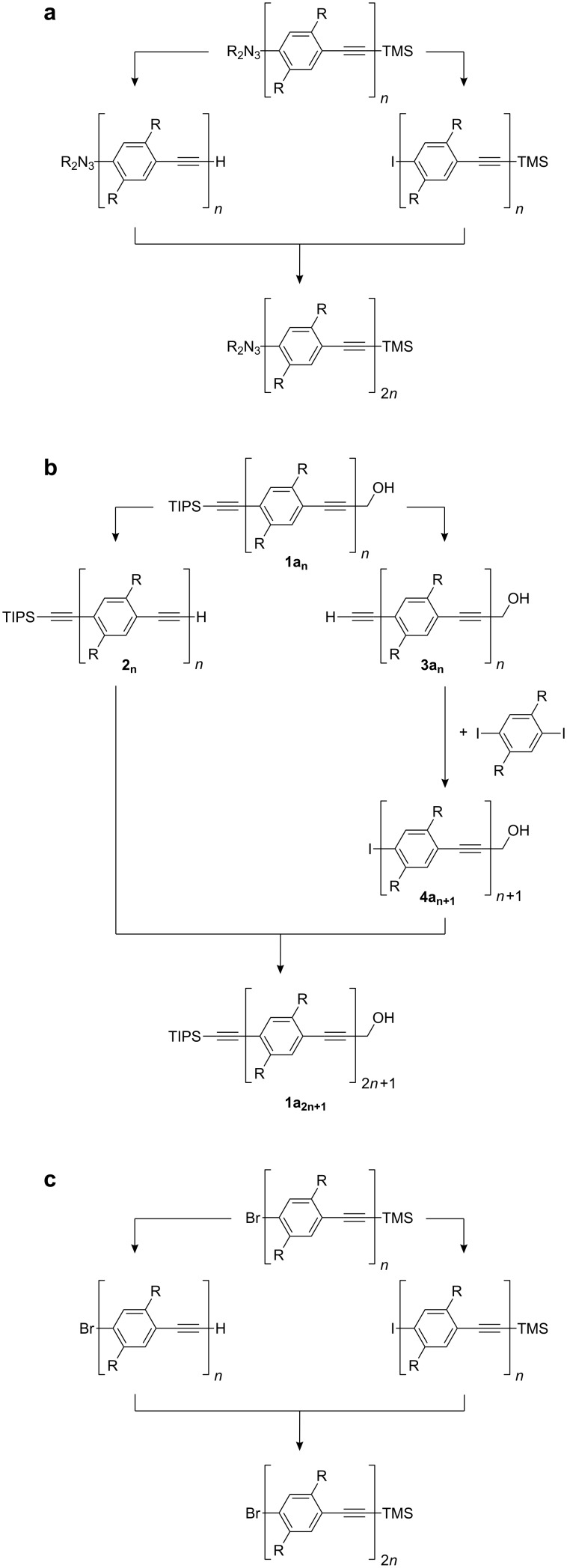
Three divergent-convergent routes to oligoPPEs. R denotes solubilising substituents such as hexyl.

Oligo(*p*-phenyleneethynylene)s (oligoPPEs) have been frequently used as structural units for nanoscopic molecules because of their geometry and their electronic and photophysical properties [1–19]. For their preparation three widely used synthetic routes have emerged:

1. The repeating unit by repeating unit approach (Scheme 1a) with desilylation and coupling of the (functionalized) arylethyne with 1-iodo-4-(2-trimethylsilylethynyl)benzene as the two repeating steps [6,9,20–24]. The oligomer grows slowly repeating unit by repeating unit. In the related bidirectional approach two repeating units are added in each coupling step (Scheme 1b) [25].
2. The divergent-convergent Moore–Tour-route (Scheme 2a) [26–29] which employs the diethyltriazenyl group to mask an iodo substituent [30–31]. 1-(Diethyltriazenyl)-4-(2-trimethylsilylethynyl)benzene is the parent compound. Desilylation and exchange of the triazenyl substituent for an iodo substituent are the two divergent steps followed by the alkynyl–aryl coupling, the convergent step. The dialkyltriazenyl group decomposes during chromatography on silica gel [28].
3. The divergent-convergent route which makes use of the orthogonality of the two alkyne protecting groups triisopropylsilyl (TIPS) and hydroxymethyl (HOM) (Scheme 2b) [32]. The reaction sequence starts with the HOM and TIPS protected 1,4-diethynylbenzene **1a_1_** from which the monoprotected 1,4-diethynylbenzenes **2_1_** and **3a_1_** are derived by the removal of either the HOM or the TIPS group. The HOM protected 1,4-diethynylbenzene **3a_1_** is coupled with 1,4-diiodobenzene to obtain aryl iodide **4a_2_**. This is coupled with the TIPS protected 1,4-diethynylbenzene **2_1_** in the convergent step. It has been shown that HOM can be exchanged for 1-hydroxy-1-methylethyl (2-hydroxyprop-2-yl, HOP) [33–38].

A rather rarely utilized third divergent-convergent approach (Scheme 2c) [39–41] relies on the bromo iodo selectivity of the alkynyl–aryl coupling and bromo iodo exchange via halogen metal exchange.

The principles underlying these methods have been applied to building blocks with additional substituents including functional groups as well as to other aromatic building blocks, such as biphenyl [33], bipyridine [36,42], thiophene [36,43–44], fluorene [45], and triptycene [46], and other shapes, e.g. starlike compounds [2,7,12,34,37].

The divergent-convergent route that employs the two orthogonal alkyne protecting groups TIPS and HOM (Scheme 2b) does not only allow the fast growth of oligomers – only four steps for doubling the number of repeating units with two of the four steps being experimentally extremely simple – but is especially satisfying because of a trouble-free separation of the desired alkynyl–aryl coupling product and the accompanying oxidative alkyne dimerization product (Glaser coupling product). In our experience, under the standard coupling conditions – i.e. Pd(PPh_3_)_2_Cl_2_, CuI, piperidine, THF, room temperature – Glaser coupling is much faster than the alkynyl–aryl coupling. Therefore, even traces of oxygen in the reaction vessel will lead to alkyne dimerization. Furthermore, most experimentalists prefer to work up the reaction mixtures under standard conditions which means exposing the reaction mixture to air. Opening the flask will immediately cause any unreacted terminal alkyne to undergo Glaser coupling. This is of no concern provided the alkyne dimer and the alkynyl–aryl coupling product can be easily separated, and this is what the HOM and related HOP group guarantee since they act as polar tags for the chromatographic separation. Polar tagging with HOM [47–49] or HOP [34,42,45,50–57] has been the key to the successful syntheses of a variety of aryleneethynylene building blocks and oligomers [42,45,47–52] and of oligoeneynes [53] including the natural marine compound callyberyne [54].

Since we disclosed this strategy several years have elapsed during which time we have gained more experience with it, became aware of some problems concerning it and improved it. Because the strategy has been adopted in whole or in part by other groups [33–34,47,49,58–59] and oligoPPEs are still a topic of great interest [1–19], we would like to share our results and present an update and an extension of our route in this paper. There are four issues that we want to address: (1) The type of MnO_2_ used for the removal of the HOM group, (2) carbometalation, a side reaction when using hydroxymethyl as an alkyne protecting group, (3) purity of 1,4-dihexyl-2,5-diiodobenzene, and (4) polar tags in the side chains of building blocks to reduce the number of steps in oligomer synthesis.

## Results and Discussion

### Type of MnO_2_ used for alkyne deprotection

The original paper on the oxidation-decarbonylation of HOM-protected alkynes [60–61] through treatment with MnO_2_ and powdered KOH does not contain any details about the type of MnO_2_. We applied this method to the synthesis of oligoPPEs, only exchanging benzene for diethyl ether, and obtained satisfactory results with commercially available active MnO_2_ (Aldrich) [32]. However, the deprotection of the HOM-protected arylalkynes **1a_n_** took several hours, especially when the reaction was run on larger scale, i.e. with 500 mg or more of starting material [62]. Even more annoying was that the required reaction time varied drastically from one experiment to another. The best procedure was to add portions of a mixture of MnO_2_ and KOH in intervals of 15 to 60 minutes until the reaction was complete. The reaction can be easily monitored by thin layer chromatography.

To improve the procedure, we tested the activated MnO_2_ (purchased; Aldrich), highly activated MnO_2_ (self-made) [63–65], BaMnO_4_ (purchased) [66–68], and γ-MnO_2_ (self-made) [63–64] on 3-(4-bromophenyl)prop-2-ynol in the presence of powdered KOH in diethyl ether at room temperature. The reaction with γ-MnO_2_ was the fastest. Even more important, γ-MnO_2_ when applied to the oligomers **1a_n_** proved to be highly reliable in its oxidizing power making it the reagent of choice for the removal of HOM groups. Some experimental results hint at a reduced activity of γ-MnO_2_ after it was stored for more than half a year in a closed jar under ambient conditions.

### Carbometalation

**Scheme 3:**
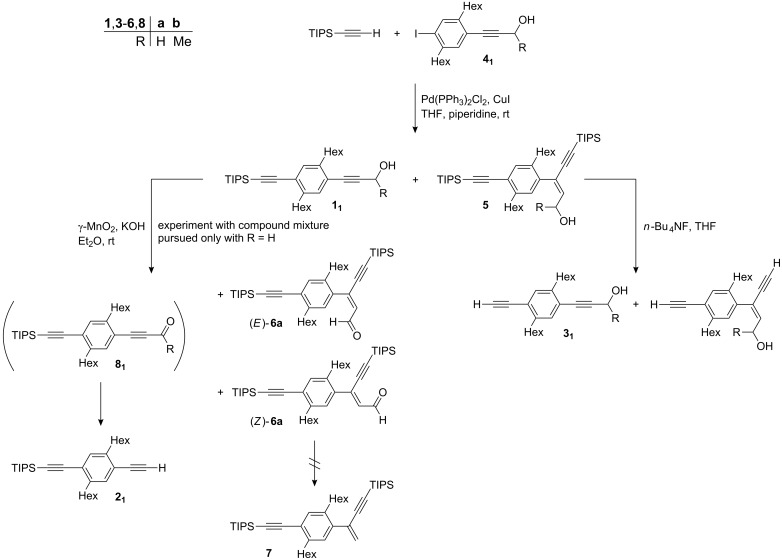
Synthesis of the building blocks **1_1_**, **2_1_**, and **3_1_**. The depicted alkene configuration of **5** was chosen assuming a carbometalation process for the formation of **5** and thus a *syn* addition of the alkyne onto the hydroxypropyne moiety.

When we published the synthesis of oligoPPEs via the divergent-convergent route which is based on the orthogonality of the alkyne protecting groups TIPS and HOM, we reported the carbometalation product **5a** (Scheme 3) which formed as a side product in the coupling of iodo monomer **4a_1_** with TIPSethyne [32]. In the original procedure a reaction temperature of 50 °C was employed. Much later we found that the reaction goes to completion even at room temperature. Lowering the temperature reduced the amount of the carbometalation product **5a** from 2–16% to 1–5%. Nevertheless, large scale preparative chromatographic separation on silica gel is tedious. Carbometalation product **5a** and monomer **1a_1_** have very similar *R*_f_-values and, unfortunately, the byproduct is eluted first.

Luckily, contamination of monomer **1a_1_** with carbometalation product **5a** is of no concern if this material is used to prepare the TIPS protected 1,4-diethynylbenzene **2_1_** (Scheme 3). Under standard reaction conditions – γ-MnO_2_, powdered KOH, Et_2_O, room temperature – the alcohol groups of both compounds **1a_1_** and **5a** are oxidized. Whereas the oxidation product of **1a_1_**, aldehyde **8a_1_**, reacts with KOH to give the ethynyl anion and formic acid which immediately exchange a proton providing deprotected alkyne **2_1_**, the oxidation product of **5a**, aldehyde **6a**, is inert under the reaction conditions [69–70]. This finding is attributed to the higher energy content and therefore lower nucleofugicity of a vinyl anion as compared to an ethynyl anion. The products, alkyne **2_1_** and the oxidized carbometalation product **6a**, are easily separable by chromatography, which resembles a simple filtration through silica gel because **6a** stays anchored on the solid phase through its polar carbonyl group. In this way pure alkyne **2_1_** can be obtained even if carbometalation product **5a** is present in the starting material.

**Scheme 4:**
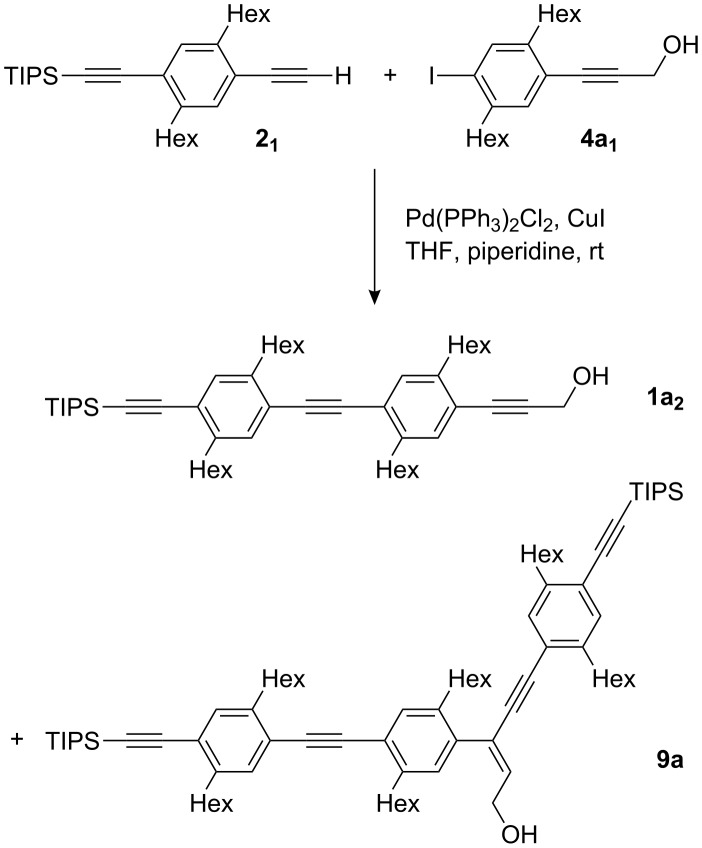
Carbometalation, an occasionally detected side reaction. The depicted alkene configuration was chosen assuming a carbometalation process and thus a *syn* addition of the alkyne onto the hydroxypropyne moiety.

The case is quite different when monomer **1a_1_** is used as the precursor for the HOM protected 1,4-diethynylbenzene **3a_1_**. Treatment of a mixture of **1a_1_** and **5a** with *n*-Bu_4_NF will not only remove the TIPS group of **1a_1_** but also the two TIPS groups of **5a** (Scheme 3). The two products are as difficult to separate as the starting compounds. Furthermore, the ethynyl groups of both products are expected to have the same reactivity which can make the isolation of pure compounds of subsequent coupling reactions even more challenging. Finally, we found that carbometalation not only occurs during the preparation of monomer **1a_1_** and its trimethylsilyl (TMS)-analogue but also, even though extremely rarely, at later stages of the oligoPPE synthesis. For example, on one occasion we isolated compound **9a** (2%, isolated yield) from the reaction between alkyne **2_1_** and iodo monomer **4a_1_** which gave dimer **1a_2_** as the major product (79%, isolated yield) (Scheme 4).

In other reactions of this type, the carbometalation product may have remained undetected due to the limited sensitivity of ^1^H NMR spectroscopy, which we use as a routine method to assess the composition of the crude product and the chromatographic fractions, although the characteristic triplet at 6.38 ppm (*J* = 7 Hz) [71] arising from the vinyl proton of the carbometalation products is easily observed. If the ^1^H NMR spectrum displays the carbon satellites of an aromatic proton signal from the major product, the threshold for detection the carbometalation product is as low as 0.5%.

While compiling records on carbometalation of hydroxymethyl protected arylalkynes we never found any evidence for carbometalation of TMS or TIPS protected arylalkynes, even when conducting the aryl–alkyne coupling at 50 °C [72–75]. Possibly, the OH-group of the HOM group coordinates to the alkyne loaded Pd(II)-complex and thus acts as a directing and rate increasing group. It may as well be that the bulky trialkylsilyl substituents simply act as steric shields for the arylalkyne. If the latter is true, then the use of 1-hydroxyethyl (HOE) or HOP instead of HOM as polar protecting groups for alkynes could prevent carbometalation. Although HOP is a sterically more demanding group, we decided in favor of HOE because the removal of HOP requires refluxing in toluene for several hours in the presence of sodium hydride or potassium hydroxide [34,36–38,42,50–52,76] whereas we expected that HOE could be detached through treatment with γ-MnO_2_ and powdered KOH in diethyl ether at room temperature, i.e. under the same, comparatively mild reaction conditions that had been used for HOM protected alkynes.

For a comparison of the influence of HOM and HOE on the carbometalation, the iodo monomers **4a_1_** and **4b_1_** were coupled with TIPSethyne in THF and piperidine using Pd(PPh_3_)_2_Cl_2_ and CuI as the catalysts. A reaction temperature of 40 °C was chosen in order to boost carbometalation. These two experiments were carried out at the same time, thus providing the best basis for a comparison. After an aqueous workup, the reaction products were analyzed by ^1^H NMR spectroscopy. In both cases the conversion of **4_1_** was complete and the main component of the crude product was the coupling product **1_1_**. The spectra gave no indication of the presence of carbometalation product **5b** whereas carbometalation product **5a** (ca. 3%) had formed. However, when another member of our group performed the coupling of TIPSethyne with HOE protected iodo monomer **4b_1_**, he found a trace of carbometalation product **5b** (1%; as determined by ^1^H NMR spectroscopy; the characteristic signal is the doublet at 6.15 ppm with *J* = 9 Hz in CDCl_3_ which is assigned to the vinyl proton) in his crude product, although the reaction had been performed at room temperature. The same co-worker generally obtains a comparatively large amount of carbometalation product. Thus, the extent to which carbometalation occurs varies with the operator. So far we have no clue what the relevant factor is. The conclusion is that the alkyne protecting group HOE reduces the amount of accompanying carbometalation product when compared with HOM, however it does not inhibit it completely.

As expected, the HOE group is as smoothly removed as the HOM group through treatment of the protected alkynes **1b_n_** with γ-MnO_2_ and powdered KOH in diethyl ether at room temperature. This is illustrated for the conversion of hexamer **1b_6_** and heptamer **1b_7_** into the alkynes **2_6_** (98% isolated yield) and **2_7_** (90% isolated yield), respectively.

### Iodination of 1,4-dihexylbenzene

**Scheme 5:**
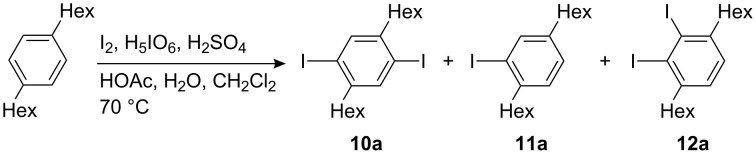
Iodination of 1,4-dihexylbenzene.

The preparation of 1,4-dihexyl-2,5-diiodobenzene (**10a**) by the iodination of 1,4-dihexylbenzene with a mixture of iodine and potassium iodate in HOAc, H_2_SO_4_, and water at 70 °C [32,77–78] gave variable yields and on occasions failed. We obtained **10a** much more reliably via iodination with iodine and periodic acid in HOAc, H_2_SO_4_, water, and dichloromethane at 70 °C [79–80]. Dichloromethane probably acts as a phase compatibiliser. Nevertheless, the reaction never went to completion: In all cases monoiodination product **11a** was found (Scheme 5). Additionally, irrespective of which procedure was followed, the crude product always contained 1,4-dihexyl-2,3-diiodobenzene (**12a**) (ca. 3%), a constitutional isomer of **10a**. The structure elucidation of these byproducts based on ^1^H NMR spectra is outlined in Supporting Information File 1.

At least twofold recrystallization is needed to obtain material which contains less than 0.5% of these byproducts (as determined by ^1^H NMR spectroscopy. The ^13^C-satellites of the signal of the aromatic protons of **10a** were used as the reference). Whereas monoiodination product **11a** is of minor concern because it is monofunctional, the constitutional isomer **12a** is a severe threat to the structural purity of oligoPPEs and especially polyPPEs. To illustrate this point let us assume that **10a** and thereof derived 1,4-diethynyl-2,5-dihexylbenzene, both contaminated with only 0.1% of the respective constitutional isomers, are polymerized to give a polymer batch with an average polymerisation degree of 1000. The consequence is that on average each of the polymer chains in this sample will have a kink, i.e. a severe structural defect.

### Polar tags in the side chain

**Scheme 6:**
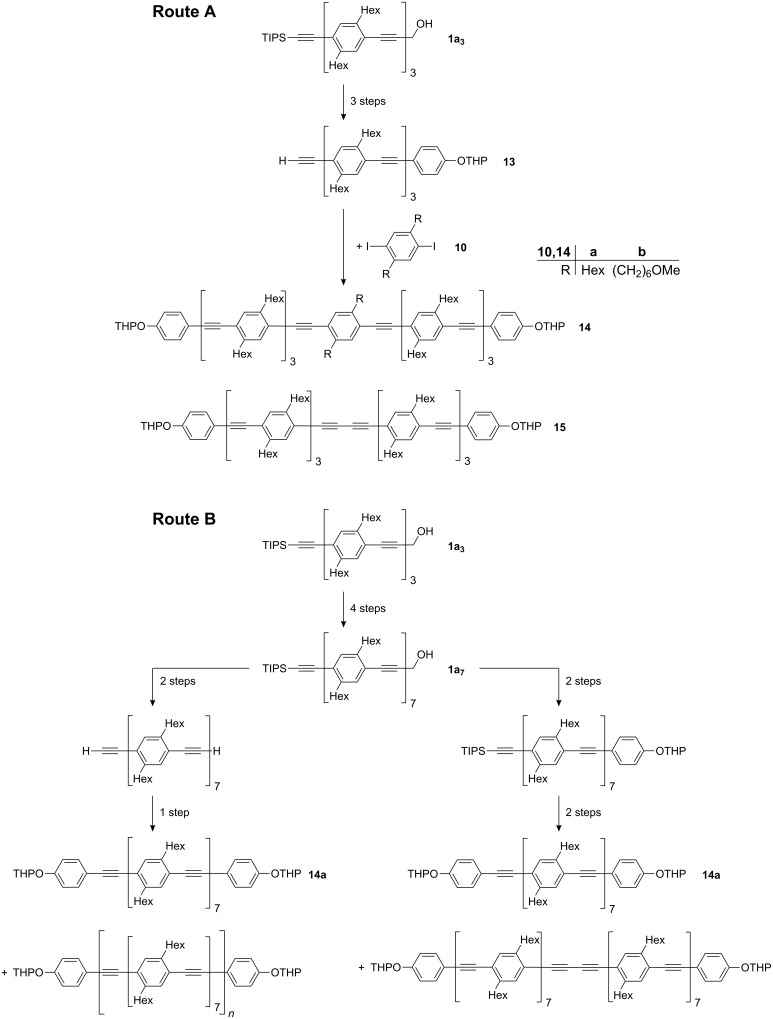
Different routes to compound **14**, a representative of the large group of functionalized oligoPPEs.

In spite of its efficiency, the divergent-convergent synthesis of oligoPPEs involves considerable effort, especially as a result of the chromatography which is required after each alkynyl–aryl coupling. In the case of the synthesis of oligoPPEs with terminal functional groups, it is tempting to reduce the number of steps through the coupling of a diiodo compound with oligoPPEs which carry one functional group and have about half of the number of repeating units of the target compound. To give one concrete example (Scheme 6): Starting from **1a_3_** the synthesis of **14a** through the coupling of **13** with diiodobenzene **10a** (Scheme 6, route A) requires only two alkynyl–aryl couplings (four steps overall), whereas the alternative (Scheme 6, route B) via heptamer **1a_7_** would take three or four cross coupling reactions (seven or eight steps overall) [81–82].

However, all of these routes will be plagued by the difficulty in separating **14a** from the accompanying alkyne dimer (Glaser coupling product). These two products differ only in the number of repeating units. In our experience with functionalized oligoPPEs their chromatographic properties on silica gel are very weakly influenced by the number of the non polar repeating unit, 2,5-dihexyl-1,4-phenyleneethynylene, but dominated by the polar functional groups. Therefore, if the two products differ in the number of polar groups, chromatographic separation can become easy. This idea was put to the test for the shortest route, route A, by employing methoxyhexyl substituted diiodobenzene **10b** instead of hexyl substituted diiodobenzene **10a**. The two methoxy groups influence the chromatographic behaviour distinctly (*R*_f_(**14b**) = 0.29, *R*_f_(**15**) = 0.71; silica gel, CH_2_Cl_2_/*n*-pentane 6:4). The oxygen atoms are intentionally inserted remote from the polyconjugated backbone in order not to change the optical properties of the oligomers.

Polar tagging with e.g. the rather inert ether moiety within the side chains at a site distant from the backbone appears to us a generally useful concept for the synthesis of mesoscopic molecules which very often have unbranched or slightly branched alkyl substituents present for solubility reasons.

## Supporting Information

Supporting information features the syntheses of compounds used for the discussed experiments, the detailed experimental procedures, and the structure elucidation of the products from the iodination of 1,4-dihexylbenzene.

File 1
