# Supplementary material for: Polar tagging in the synthesis of monodisperse oligo(p-phenyleneethynylene)s and an update on the synthesis of oligoPPEs
Source: Beilstein J Org Chem. 2010 Jun 1;6:57. doi: 10.3762/bjoc.6.57 (PMC2900939; doi:10.3762/bjoc.6.57)
Supplement: File 1 [file Beilstein_J_Org_Chem-06-57-s001.pdf]

Supporting Information

for

Polar tagging in the synthesis of monodisperse oligo(*p*-phenyleneethynylene)s and an update on the synthesis of oligoPPEs

Dhananjaya Sahoo, Susanne Thiele, Miriam Schulte, Navid Ramezanian and Adelheid Godt*

Bielefeld University, Faculty of Chemistry

Universitätsstr. 25, D-33615 Bielefeld, Germany

Email: Adelheid Godt - godt@uni-bielefeld.de

*Corresponding author

Structure elucidation of the byproducts formed on iodination of 1,4-dihexylbenzene

The structure elucidation is based on data extracted from the ^{1}H NMR spectra of compound mixtures, i.e. of crude products, crystallized material, and residues in mother liquors. Because we have ^{1}H NMR spectra of numerous mixtures with largely varying ratios of the different compounds at hand, we are able to assign the signals of the aromatic and the benzylic protons to individual compounds and thus propose the structures of monoiodination product **11a** and of 1,4-dihexyl-2,3-diiodobenzene (**12a**), a diiodination product which is a constitutional isomer of the main product **10a**. The monoiodination product **11a** gives rise to multiplets at 7.62 (1 H), 7.07-7.03 (2 H), 2.65 (2 H), and 2.50 (2 H) ppm. The diiodination product **12a** is detected through its singlet at 7.10 ppm (2 H) and a triplet like multiplet at 2.85 ppm (4 H) ($CDCl_3$, room temperature, 250 MHz). As shown in table S1, the ^{1}H NMR data calculated for the diiodination product **12a** from the reported data of 2,3-diiodo-1,4-dimethylbenzene [1], the methyl analogue of **12a**, are in good agreement with those found.

The formation of the byproducts is not unique for the iodination of 1,4-dihexyl-benzene. Corresponding byproducts were formed upon iodination of 1,4-bis(6-methoxyhexyl)benzene and 1,4-dimethylbenzene.

Table S1: Comparison of calculated and found ^{1}H NMR data of **12a** (in ppm, $CDCl_3$, room temperature). The signal positions for **12a** were calculated with the help of the reported ^{1}H NMR data of 2,3-diiodo-1,4-dimethylbenzene [1] and increments for the transition from the 1,4-dimethyl to the 1,4-dihexyl substituted benzene. These increments were extracted from the ^{1}H NMR data of **10a** [3] and its methyl analogue 1,4-diiodo-2,5-dimethylbenzene [2]. The observed data correspond well with the calculated data for **12a**.

	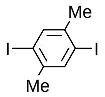	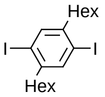 **10a**	increments for the transition Me → Hex	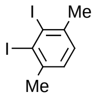	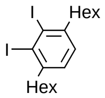 **12a**	
					calc.	found
ArC<u>H</u>$_2$	2.33	2.58	+ 0.25	2.5	2.75	2.85
Ar<u>H</u>	7.64	7.59	− 0.05	7.1	7.05	7.10

Building blocks for oligoPPEs with HOE instead of HOM as the alkyne protecting group

The basic building blocks iodo monomer **4b$_1$**, monomer **1b$_1$**, its TMS analogue **17b$_1$**, polar alkyne **3b$_1$**, and iodo dimer **4b$_2$** were obtained using the procedures that have been developed for the corresponding HOM protected compounds (Scheme S1). The polar alkyne **3b$_1$** can be prepared either from monomer **1b$_1$** or from the TMS analogue **17b$_1$**. The latter is preferred because it is the cheaper starting material and the removal of the TMS group requires only inexpensive reagents.

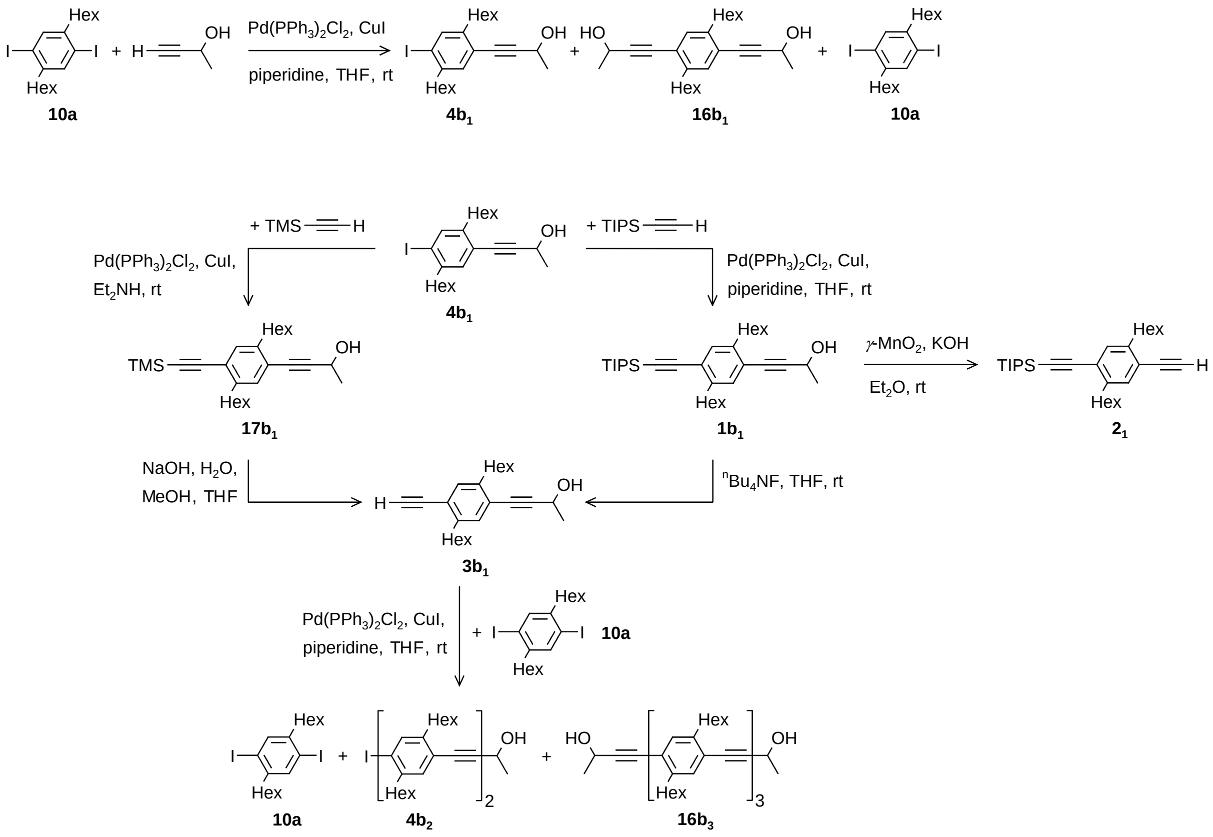

Scheme S1: Synthesis of building blocks for the preparation of oligoPPEs using HOE as the alkyne protecting group.

Experimental procedures

General: All reactions, except the iodination, were performed under an argon atmosphere. Solvents were removed under reduced pressure at a bath temperature of ca. 40 ^{o}C. THF was distilled from sodium/benzophenone. Piperidine was distilled from CaH_2. Et_2NH was used as received.

Merck silica gel (40-63 µm) was used for column chromatography. The size of the silica gel column is given in diameter x length. If not otherwise noted, the material was dissolved in a minimum quantity of CH_2Cl_2 and the solution obtained poured on top of the silica gel column. In most cases, a slight pressure was applied during chromatography. The compositions of solvent mixtures are given in volume ratios. Thin layer chromatography (TLC) was carried out on silica gel coated aluminum foil (Merck, 60 F_{254}).

The melting points were determined in open capillaries. Unless otherwise specified, NMR spectra were recorded at room temperature. The solvent was used as an internal standard. For signal assignment, the type of carbon atom - quaternary, tertiary, secondary, primary - was determined by a DEPT 135 experiment. 1,8,9-Trihydroxyanthracene (dithranol) was used as the matrix for MALDI-TOF experiments.

The alkynes **2_2** and **2_4** were obtained as described [3], however *γ*-MnO_2 was employed instead of activated MnO_2. 1,4-Dihexylbenzene [4], dimer **$1a_2$** [3], and compound **13** [5] were synthesized according to the literature procedures.

***γ*-MnO_2** [6]**.** A solution of $KMnO_4$ (26.27 g, 0.17 mol) in water (500 mL) was added to a hot (60 ^oC) solution of $MnSO_4 \bullet H_2O$ (42.26 g, 0.25 mol) in water (750 mL) over the course of half an hour. The suspension was stirred at 60 ^oC for another 2 h before the solid was isolated, carefully washed with water (12 times with 15 mL), and finally dried to constant weight (41.6 g of dark brown solid) at 60 ^oC in an oven.

New procedure for the preparation of 1,4-dihexyl-2,5-diiodobenzene (10a). Iodine (26.0 g, 102 mmol), H_5IO_6 (11.3 g, 49.7 mmol), and concentrated sulfuric acid (10.5 mL) were added to the biphasic mixture of 1,4-dihexylbenzene (30.0 g, 122 mmol), CH_2Cl_2 (45 mL), H_2O (54 mL), and acetic acid (250 mL). The reaction mixture was stirred vigorously for 17.5 h at 70 ^oC. After cooling to ice bath temperature, the precipitate was isolated, washed well with water, and dried (evacuated dessicator with P_4O_{10}). The solid consisted of 1,4-dihexyl-2,5-diiodobenzene (**10a**), 1,4-dihexyl-2-iodobenzene (**11a**), and 1,4-dihexyl-2,3-diiodobenzene (**12a**) in the ratio of 26:10:1 (as determined by ^1H NMR spectroscopy). It was poured onto a silica gel column (4 cm x 6 cm). Elution with *n*-pentane (ca. 500 mL), subsequent removal of *n*-pentane

from the eluate, and finally twofold recrystallization of the residue from ethanol (first time: 200 mL, second time: 150 mL) gave 1,4-dihexyl-2,5-diiodobenzene (**10a**; 26 g, 43 %) containing traces of 1,4-dihexyl-2,3-diiodobenzene (**12a**) and 1,4-dihexyl-2-iodobenzene (**11a**) (less than 0.5 % as determined by ^{1}H NMR spectroscopy) as colorless needles. NMR data are identical to those reported earlier [3]. Anal. Calcd for $C_{18}H_{28}I_2$ (498.232 g/mol): C, 43.39; H, 5.66. Found C, 43.43; H, 5.58.

Alkynyl-aryl coupling (Sonogashira-Hagihara coupling) - general procedure

The solution of the two coupling components in a mixture of dry THF and dry piperidine was degassed through several freeze-pump-thaw-cycles. The solution was still very cold when a mixture of $Pd(PPh_3)_2Cl_2$ and CuI was added. Shortly after, a voluminous precipitate formed. The reaction mixture was stirred at room temperature, then diethyl ether and finally water were added. The phases were separated and the aqueous phase was extracted with Et_2O. The combined organic phases were washed with 2N HCl and then with saturated aqueous NaCl. After drying the organic phase with $MgSO_4$ or Na_2SO_4, the solvents were removed and the residue was fractionated by column chromatography.

Removal of the hydroxymethyl (HOM) or hydroxyethyl (HOE) group - general procedure

To a solution of oligoPPEs $\mathbf{1a_n}$ or $\mathbf{1b_n}$ in dry diethyl ether, a mixture of γ-MnO_2 and powdered KOH was added portionwise, one portion every half an hour or hour. The suspension was stirred with protection from light at room temperature. The reaction can be monitored by TLC (CH_2Cl_2/*n*-pentane 1:1). Filtration of the reaction mixture through a short column of silica gel with diethyl ether gave, after evaporation of the solvent, the non-polar alkynes $\mathbf{2_n}$.

New procedure for the preparation of monomer $\mathbf{1a_1}$. See also the general procedure for alkynyl-aryl coupling. Iodo monomer $\mathbf{4a_1}$ (3.30 g, 7.74 mmol) was coupled with triisopropylsilylethyne (1.9 mL, 8.5 mmol) in THF (40 mL) and piperidine (10 mL) mediated by $Pd(PPh_3)_2Cl_2$ (54 mg, 0.08 mmol) and CuI (30 mg, 0.16 mmol). Reaction time: 19 h. The crude product contained around 1% of carbometalation product **5a** (as determined by ^1H NMR spectroscopy taking the intensities of the ^{13}C satellites of the signals of the aromatic protons of $\mathbf{1a_1}$ as a reference.). Column chromatography (the crude product was dissolved in *n*-pentane/CH_2Cl_2 1:2; 4.5 cm x 27 cm; *n*-pentane/CH_2Cl_2 1:2) yielded $\mathbf{1a_1}$ (3.19 g, 86%; R_f = 0.40) as an orange-brown oil which was preceded by a fraction containing carbometalation product **5a** (R_f = 0.49), $\mathbf{1a_1}$, and 1,2-bis(triisopropylsilyl)butadiyne. For analytical data see ref. [3].

Procedure for the preparation of alkyne $\mathbf{2_1}$ from a mixture of monomer $\mathbf{1a_1}$ and carbometalation product 5a. When applying the published procedure for the preparation of alkyne $\mathbf{2_1}$ [3] to a mixture (400 mg) of monomer $\mathbf{1a_1}$ and carbometalation product **5a** in a ratio of about 13:1, a mixture of alkyne $\mathbf{2_1}$ and aldehyde **6a** was obtained. Column chromatography (the crude product was dissolved in *n*-pentane/CH_2Cl_2 5:1; *n*-pentane/CH_2Cl_2 5:1) gave alkyne $\mathbf{2_1}$ (296 mg, 79%; R_f = 0.63) as a yellow-orange oil. The aldehyde **6a** (R_f = 0.10) was not eluted. For analytical data see ref. [3].

Basic building blocks for oligoPPEs $\mathbf{1b_n}$ with HOE instead of HOM as the alkyne protecting group

Iodo monomer $\mathbf{4b_1}$. See also the general procedure for alkynyl-aryl coupling. 3-Butyn-2-ol (580 µL, 7.4 mmol) and 1,4-dihexyl-2,5-diiodobenzene (**10a**) (5.49 g,

11.02 mmol) were coupled in THF (70 mL) and piperidine (15 mL) using $Pd(PPh_3)_2Cl_2$ (52 mg, 0.07 mmol) and CuI (28 mg, 0.15 mmol) as the catalysts Reaction time: 17.5 h. Column chromatography (the crude product was dissolved in Et_2O/*n*-pentane 2:5; 4 cm x 35 cm; Et_2O/*n*-pentane 2:5) gave diiodobenzene **10a** (2.63 g, 48%; R_f = 0.71) as a yellow solid, iodo monomer $\mathbf{4b_1}$ (2.17 g, 67%; R_f = 0.31) as a slowly crystallizing yellow oil, and the dicoupling product $\mathbf{16b_1}$ (369 mg, 26%; R_f = 0.11) contaminated with a trace of unidentified compounds (^{1}H NMR signals of very low intensity between 7.8 and 7.3 ppm) as an orange solid.

Analytical data of iodo monomer $\mathbf{4b_1}$: mp 33-34 °C; ^{1}H NMR (250 MHz, CD_2Cl_2): δ = 7.66 (s, 1 H, ArH ortho to iodo), 7.22 (s, 1 H, ArH meta to iodo), 4.75 (m, 1 H, $\mathrm{C\underline{H}OH}$), 2.68-2.60 (m, 4 H, $\mathrm{ArC\underline{H}_2}$), 2.03 (d, J = 5.3 Hz, 1 H, OH), 1.65-1.50 (m, 4 H, $\mathrm{ArCH_2C\underline{H}_2}$), 1.53 (d, J = 6.6 Hz, 3 H, $\mathrm{CH(OH)C\underline{H}_3}$), 1.44-1.25 (m, 12 H, CH_2), 0.90 (m, 6 H, CH_3); ^{13}C NMR (63 MHz, CD_2Cl_2): δ = 144.6 ($\mathrm{\underline{C}Hex}$ ortho to iodo), 143.2 ($\mathrm{\underline{C}Hex}$ meta to iodo), 139.8 (arom. CH ortho to iodo), 132.7 (arom. CH meta to iodo), 122.6 ($\mathrm{\underline{C}C{\equiv}C}$), 101.2 (C-I), 95.8 and 82.3 ($\mathrm{\underline{C}{\equiv}\underline{C}CHOH}$), 59.2 (CHOH), 40.5 ($\mathrm{Ar\underline{C}H_2}$ ortho to iodo), 34.0 ($\mathrm{Ar\underline{C}H_2}$ meta to iodo), 32.07, 32.06, 30.9, 30.6, 29.5, and 29.4 (CH_2), 24.7 ($\mathrm{CH(OH)\underline{C}H_3}$), 23.0 ($CH_2$), 14.2 ($\mathrm{CH_2\underline{C}H_3}$); Anal. Calcd for $C_{22}H_{33}IO$ (440.410 g/mol): C, 60.00; H, 7.55. Found C, 59.80; H, 7.34.

Analytical data of dicoupling product $\mathbf{16b_1}$: mp 65-67 °C (impure sample); ^{1}H NMR (250 MHz, CD_2Cl_2): δ = 7.22 (s, 2 H, ArH), 4.77 (m, 2 H, $\mathrm{C\underline{H}OH}$), 2.68 (t-shaped signal, J = 7.6 Hz, 4 H, $\mathrm{ArC\underline{H}_2}$), 2.23 (s, 2 H, OH), 1.60 (m, 4 H, $\mathrm{ArCH_2C\underline{H}_2}$), 1.54 (d, J = 6.6 Hz, 6 H, $\mathrm{CH(OH)C\underline{H}_3}$), 1.45-1.25 (m, 12 H, CH_2), 0.90 (m, 6 H, CH_3); ^{13}C NMR (63 MHz, CD_2Cl_2): δ = 142.7 ($\mathrm{\underline{C}Hex}$), 132.7 (arom. CH), 122.5 ($\mathrm{\underline{C}C{\equiv}C}$), 96.1 and 82.7 ($\mathrm{\underline{C}{\equiv}\underline{C}CHOH}$), 59.2 ($\mathrm{\underline{C}HOH}$), 34.3 ($\mathrm{Ar\underline{C}H_2}$), 32.1, 31.9, 30.9, 29.8, and 29.5 (CH_2), 24.8 ($\mathrm{CH(OH)\underline{C}H_3}$), 23.0 ($CH_2$), 14.3 ($\mathrm{CH_2\underline{C}H_3}$).

Monomer $1b_1$. See also the general procedure for alkynyl-aryl coupling. Iodo monomer **$4b_1$** (2.05 g, 4.65 mmol) and triisopropylsilylethyne (1.15 mL, 5.12 mmol) were coupled in THF (25 mL) and piperidine (6 mL) using $Pd(PPh_3)_2Cl_2$ (33 mg, 0.05 mmol) and CuI (18 mg, 0.09 mmol) as the catalysts. Reaction time: 18.5 h. Column chromatography (the crude product was dissolved in Et_2O/*n*-pentane 3:7; 3 cm x 25 cm; Et_2O/*n*-pentane 3:7) afforded a mixture (885 mg) of monomer **$1b_1$** and carbometalation product **5b** (R_f = 0.63), and a fraction of TLC pure monomer **$1b_1$** (1.25 g, 54%; R_f = 0.56). The mixture was further fractionated by column chromatography (the mixture was dissolved in Et_2O/*n*-pentane 3:7; 3 cm x 28 cm; Et_2O/*n*-pentane 3:7) to give a mixture (290 mg) of monomer **$1b_1$** and carbometalation product **5b** in a ratio of ca. 94:6 (as determined by ^{1}H NMR spectroscopy) and TLC pure monomer **$1b_1$** (575 mg, 25%). Monomer **$1b_1$** (1.82 g, 79%) was obtained as an orange oil. ^{1}H NMR (500 MHz, CD_2Cl_2): δ = 7.27 and 7.22 (2 s, 1 H each, ArH), 4.76 (m, 1 H, CHOH), 2.73 and 2.68 (2 t-shaped signals, J = 8 Hz, 2 H each, $ArCH_2$), 2.01 (d, J = 5.2 Hz, 1 H, OH), 1.60 (m, 4 H, $ArCH_2CH_2$), 1.53 (d, J = 6.4 Hz, 3 H, $CH(OH)CH_3$), 1.39-1.28 (m, 12 H, CH_2), 1.14 (s, 21 H, TIPS), 0.89 (m, 6 H, CH_3). ^{13}C NMR (126 MHz, CD_2Cl_2): δ = 143.0 and 142.6 (CHex), 133.1 and 132.7 (arom. CH), 123.2 and 122.3 (CC≡C), 105.9 (C≡CTIPS), 96.1 (C≡CCHOH), 95.6 (C≡CTIPS), 82.7 (C≡CCHOH), 59.2 (CHOH), 34.7 and 34.3 ($ArCH_2$), 32.2, 32.1, 31.3, 31.0, 29.7, and 29.6 (CH_2), 24.7 ($CH(OH)CH_3$), 23.0 (CH_2), 18.8 ($CH(CH_3)_2$), 14.2 (CH_2CH_3), 11.7 (SiCH): Anal. Calcd for $C_{33}H_{54}OSi$ (494.880): C, 80.09; H, 11.00. Found C, 79.92; H, 10.95.

Compound $17b_1$, the TMS analogue of monomer $1b_1$. To a degassed (several freeze-pump-thaw cycles) solution of iodo monomer **$4b_1$** (1.67 g, 3.79 mmol) and trimethylsilylethyne (0.59 mL, 4.2 mmol) in Et_2NH (30 mL), $Pd(PPh_3)_2Cl_2$ (27 mg, 0.04 mmol) and CuI (14 mg, 0.07 mmol) were added. Shortly after, a second liquid phase

formed. After stirring the reaction mixture for 16 h at room temperature, Et_2NH was removed under slightly reduced pressure. Diethyl ether and then water were added to the residue. The phases were separated and the aqueous phase was extracted with Et_2O. The combined organic phases were washed with saturated aqueous NH_4Cl. After drying the organic phase with Na_2SO_4, the solvents were removed and compound **$17b_1$** isolated by column chromatography (the crude product was dissolved in *n*-pentane/CH_2Cl_2 1:2; 3 cm x 30 cm; *n*-pentane/CH_2Cl_2 1:2) as an orange oil (1.39 g, 90%; R_f = 0.33). ^{1}H NMR (250 MHz, CD_2Cl_2): δ = 7.25 (s, 1 H, ArH), 7.23 (s, 1 H, ArH), 4.77 (m, 1 H, $C\underline{H}OH$), 2.73-2.65 (m, 4 H, $ArC\underline{H_2}$), 2.12 (d, J = 5.0 Hz, 1 H, OH), 1.61 (m, 4 H, $ArCH_2C\underline{H_2}$), 1.54 (d, J = 6.6 Hz, 3 H, $CH(OH)C\underline{H_3}$), 1.40-1.28 (m, 12 H, CH_2), 0.90 (m, 6 H, CH_3), 0.27 (s, 9 H, TMS); ^{13}C NMR (63 MHz, CD_2Cl_2): δ = 143.2 and 142.7 ($\underline{C}$Hex), 132.79 and 132.77 (arom. CH), 123.0 and 122.6 ($\underline{C}C{\equiv}C$), 104.2 and 99.3 ($\underline{C}{\equiv}\underline{C}TMS$), 96.3 and 82.8 ($\underline{C}{\equiv}\underline{C}CHOH$), 59.2 (CHOH), 34.5 and 34.3 ($Ar\underline{C}H_2$), 32.2, 31.04, 30.97, 29.7, and 29.6 (CH_2), 24.8 ($CH(OH)\underline{C}H_3$), 23.09 and 23.06 (CH_2), 14.3 ($CH_2\underline{C}H_3$), 0.1 ($Si(CH_3)_3$); Anal. Calcd for $C_{27}H_{42}OSi$ (410.718 g/mol): C, 78.96; H, 10.31. Found C, 78.91; H, 10.40.

HOE protected 1,4-diethynylbenzene $3b_1$ starting from $17b_1$. Aqueous 5N NaOH (3.5 mL) was added to a solution of compound **$17b_1$** (1.25 g, 3.04 mmol) in MeOH (20 mL) and THF (15 mL). After two hours diethyl ether and water were added. The phases were separated and the aqueous phase extracted with diethyl ether. The combined organic phases were washed with water, then with saturated aqueous NaCl, and finally dried ($MgSO_4$). Column chromatography (the crude product was dissolved in *n*-pentane/CH_2Cl_2 1:2; 3 cm x 30 cm; *n*-pentane/CH_2Cl_2 1:2) afforded HOE protected 1,4-diethynylbenzene **$3b_1$** (951 mg, 92%; R_f = 0.33) as a yellow oil. The crude product can probably be used without further purification. ^{1}H NMR (500 MHz, $CDCl_3$): δ = 7.27 (s, 1 H, ArH), 7.22 (s, 1 H, ArH), 4.77 (m, 1 H, $C\underline{H}OH$), 3.26

(s, 1 H, C≡CH), 2.67 (m, 4 H, $ArC\underline{H}_2$), 1.87 (m, 1 H, OH), 1.58 (m, 4 H, $ArCH_2C\underline{H}_2$), 1.56 (d, J = 6.5 Hz, 3 H, $CH(OH)C\underline{H}_3$), 1.36-1.26 (m, 12 H, CH_2), 0.87 (m, 6 H, CH_3).

Iodo dimer $\mathbf{4b_2}$. See also the general procedure for alkynyl-aryl coupling. HOE protected 1,4-diethynylbenzene $\mathbf{3b_1}$ (934 mg, 2.76 mmol) and 1,4-dihexyl-2,5-diiodobenzene (**10a**) (4.48 g, 8.99 mmol) were coupled in THF (40 mL) and piperidine (15 mL) with $Pd(PPh_3)_2Cl_2$ (20 mg, 0.03 mmol) and CuI (11 mg, 0.06 mmol) as the catalysts. Reaction time: 15 h. The crude product was adsorbed onto a small quantity of silica gel by dissolving it in CH_2Cl_2, adding silica gel to this solution, and removing the solvent (40 °C, reduced pressure). The resulting fine, free flowing powder was transferred onto the top of a silica gel column (4.5 cm x 31 cm). Chromatography (Et_2O/*n*-pentane 2:5) gave diiodobenzene **10a** as a yellow solid (3.28 g, 73%; R_f = 0.74), iodo dimer $\mathbf{4b_2}$ as a light-yellow solid (1.05 g, 54%; R_f = 0.34), and a 4:1 mixture of the disubstitution product $\mathbf{16b_3}$ and the oxidative dimerization (Glaser coupling) product of alkyne $\mathbf{3b_1}$ as a yellow solid (405 mg; R_f = 0.10). Analytical data of iodo dimer $\mathbf{4b_2}$: mp 50-52 °C; ^{1}H NMR (250 MHz, CD_2Cl_2): δ = 7.71 (s, 1 H, ArH ortho to iodo), 7.33, 7.32, and 7. 28 (3 s, 1 H each, ArH), 4.78 (d of q, J_q = 6.5 Hz, J_d = 5.5 Hz, 1 H, $C\underline{H}OH$), 2.83-2.64 (m, 8 H, $ArC\underline{H}_2$), 1.99 (d, J = 5.4 Hz, 1 H, OH), 1.64 (m, 8 H, $ArCH_2C\underline{H}_2$), 1.55 (d, J = 6.6 Hz, 3 H, $CH(OH)C\underline{H}_3$), 1.45-1.25 (m, 24 H, CH_2), 0.90 (m, 12 H, CH_3); ^{13}C NMR (63 MHz, CD_2Cl_2): δ = 144.3 ($\underline{C}$Hex ortho to iodo), 143.3, 142.8 and 142.4 ($\underline{C}$Hex), 139.9 (arom. CH ortho to iodo), 132.8 and 132.7 (arom. CH), 123.4, 123.2 and 122.5 ($\underline{C}$C≡C), 101.2 (C-I), 96.3 ($\underline{C}$≡$\underline{C}$CHOH), 92.7 and 92.6 (Ar$\underline{C}$≡$\underline{C}$Ar), 82.8 ($\underline{C}$≡$\underline{C}$CHOH), 59.3 (CHOH), 40.6 ($Ar\underline{C}H_2$ ortho to iodo) 34.5, 34.3 and 34.2 ($Ar\underline{C}H_2$), 32.22, 32.18, 32.14, 32.08, 31.09, 31.06, 31.0, 30.6, 29.62, 29.58, and 29.4 (CH_2), 24.8 ($CH(OH)\underline{C}H_3$), 23.0 (CH_2), 14.3 ($CH_2\underline{C}H_3$); MALDI-TOF (708.854 g/mol calcd for $C_{42}H_{61}IO$): *m/z* 708.82 [M^+], 582.94 [M^+-Iodo].

HOE protected oligoPPEs - synthesis and smooth deprotection of the HOE protected ethyne moiety

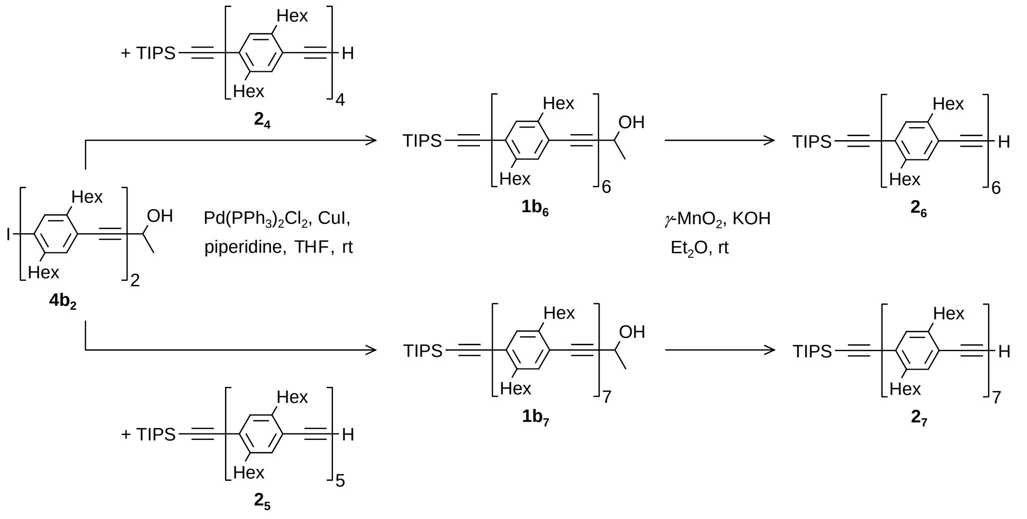

Scheme S2: Synthesis of oligoPPEs **1b$_6$** and **1b$_7$** and demonstration of the smooth removal of the HOE protecting group.

Hexamer 1b$_6$. See also the general procedure for alkynyl-aryl coupling. Iodo dimer **4b$_2$** (160 mg, 0.23 mmol) and alkyne **2$_4$** (303 mg, 0.24 mmol) were coupled in THF (13 mL) and piperidine (4 mL) with the help of $Pd(PPh_3)_2Cl_2$ (1.6 mg, 0.002 mmol) and CuI (1.3 mg, 0.007 mmol). Reaction time: 18 h. Hexamer **1b$_6$** was isolated through column chromatography (4 cm x 35 cm; *n*-pentane/CH_2Cl_2 1:1 v:v) as a yellow solid (334 mg, 81%; R_f = 0.54). The oxidative dimerization (Glaser coupling) product of alkyne **2$_4$** (38 mg, 13%; R_f = 0.96) was eluted well ahead of hexamer **1b$_6$**. Analytical data of hexamer **1b$_6$**: mp 119-121 °C; ^{1}H NMR (500 MHz, CD_2Cl_2): δ = 7.41 and 7.39 (2 s and s, respectively, 8 H, ArH), 7.35 (s, 2 H, ArH), 7.33 (s, 1 H, ArH), 7.29 (s, 1 H, ArH), 4.78 (d of q, J_q ~ J_d = 6.1 Hz, 1 H, C<u>H</u>OH), 2.86-2.80 (m, 20 H, ArC<u>H</u>$_2$), 2.78 and 2.73 (2 t-shaped signals J = 8.0 Hz, 2 H each, ArC<u>H</u>$_2$), 1.98 (broadened s, 1 H, OH), 1.76-1.61 (m, 24 H, ArCH$_2$C<u>H</u>$_2$), 1.56 (d, J = 6.2 Hz, 3 H, CH(OH)C<u>H</u>$_3$), 1.5-1.3 (m, 72 H, CH$_2$*)*, 1.16 (s, 21 H, TIPS), 0.90 (m, 36 H, C<u>H</u>$_3$); ^{13}C NMR (126 MHz, CD_2Cl_2): δ = 143.1, 142.7, 142.44, 142.42, 142.34, and 142.32

($\underline{C}$Hex), 133.2, 132.8, and 132.7 (arom. CH), 123.18, 123.15, 123.12, and 122.4 ($\underline{C}$C≡C), 106.0 ($\underline{C}$≡$\underline{C}$TIPS), 96.2 ($\underline{C}$≡$\underline{C}$CHOH), 95.8 ($\underline{C}$≡$\underline{C}$TIPS), 93.40, 93.36, 93.23, and 93.19 (Ar$\underline{C}$≡$\underline{C}$Ar), 82.8 ($\underline{C}$≡$\underline{C}$CHOH), 59.2 (CHOH), 34.7, 34.5, 34.4 and 34.3 (Ar$\underline{C}H_2$), 32.2, 32.1, 31.3, 31.2, 31.11, 31.06, 30.95, 29.72, 29.61, and 29.55 (CH_2), 24.7 (C(OH)$\underline{C}H_3$), 23.1 (CH_2), 18.8 (CH($\underline{C}H_3)_2$), 14.3 ($CH_2\underline{C}H_3$), 11.7 ($\underline{C}H(CH_3)_2$); MALDI-TOF: *m/z* 1836.53; Anal. Calcd for $C_{133}H_{194}OSi$ (1837.100 g/mol): C, 86.96; H, 10.64. Found C, 86.93; H, 10.84.

Heptamer $1b_7$. See also the general procedure for alkynyl-aryl coupling. Iodo dimer **$4b_2$** (195 mg, 0.28 mmol) and alkyne **2_5** (442 mg, 0.29 mmol; synthesis see below) were coupled in THF (12 mL) and piperidine (4 mL) using $Pd(PPh_3)_2Cl_2$ (3.0 mg, 0.004 mmol) and CuI (1.5 mg, 0.008 mmol) as the catalysts. Reaction time: 19 h. Column chromatography (4 cm x 25 cm; *n*-pentane/Et_2O, 4:1) yielded heptamer **$1b_7$** (565 mg, 97 %; R_f = 0.44) as a yellow solid. A mixture (78 mg; R_f = 93) of the oxidative dimerization (Glaser coupling) product of alkyne **2_5** and residual alkyne **2_5** was eluted well ahead of heptamer **$1b_7$**. Analytical data of heptamer **$1b_7$**: mp 133-135 °C; ^{1}H NMR (500 MHz, CD_2Cl_2): δ = 7.41, 7.40, and 7.39 (3 s, 10 H, ArH), 7.35 (s, 2 H, ArH), 7.33 (s, 1 H, ArH), 7.29 (s, 1 H, ArH), 4.78 (quint, *J* = 6.1 Hz, 1 H, C$\underline{H}$OH), 2.88-2.78 (m, 24 H, ArC$\underline{H}_2$), 2.77 (t-shaped signal, 2 H, ArC$\underline{H}_2$), 2.72 (t-shaped signal, 2 H, ArC$\underline{H}_2$), 1.96 (d, *J* = 5.4 Hz, 1 H, OH), 1.76-1.61 (m, 28 H, $ArCH_2C\underline{H}_2$), 1.55 (d, *J* = 6 Hz, 3 H, CH(OH)C$\underline{H}_3$, 1.5-1.3 (m, 84 H, CH_2), 1.16 (s, 21 H, TIPS), 0.89 (m, 42 H, CH_3); ^{13}C NMR (126 MHz, CD_2Cl_2): δ = 143.1, 142.7, 142.43, 142.41, 142.33, and 142.31 ($\underline{C}$Hex), 133.2, 132.8, and 132.7 (arom. CH), 123.16, 123.13, 123.10, and 122.3 ($\underline{C}$C≡C), 106.0 ($\underline{C}$≡$\underline{C}$TIPS), 96.2 ($\underline{C}$≡$\underline{C}$CHOH), 95.8 ($\underline{C}$≡$\underline{C}$TIPS), 93.39, 93.33, 93.21, and 93.18 (Ar$\underline{C}$≡$\underline{C}$Ar), 82.7 ($\underline{C}$≡$\underline{C}$CHOH), 59.2 ($\underline{C}$HOH), 34.7, 34.5, 34.4, and 34.3 (Ar$\underline{C}H_2$), 32.2, 32.1, 31.3, 31.2, 31.11, 31.05, 30.9, 29.71, 29.65, 29.59, and 29.54 (CH_2), 24.7 (CH(OH)$\underline{C}H_3$), 23.05 and 23.22 (CH_2), 18.8 (CH($\underline{C}H_3)_2$),

14.3 ($CH_2\underline{C}H_3$), 11.7 ($\underline{C}H(CH_3)_2$); MALDI-TOF: *m/z* = 2103.44; Anal. Calcd for $C_{153}H_{222}OSi$ (2105.544 g/mol): C, 87.28; H, 10.63. Found C, 87.28; H, 10.45.

Alkyne 2_6. See also the general procedure for HOE-removal. *γ*-MnO_2 (312 mg, 3.59 mmol) and powdered KOH (105 mg, 1.87 mmol) were added in six portions to a solution of hexamer **$1b_6$** (50 mg, 0.027 mmol) in diethyl ether (5 mL). Alkyne **2_6** (48 mg, 98%) was isolated as a yellowish solid. mp 113-114 °C; ^1H NMR (500 MHz, CD_2Cl_2): δ = 7.42, 7.41, and 7.40 (3 s, 8 H, ArH), 7.37 (s, 1 H, ArH), 7.359 and 7.357 (2 s, 2 H, ArH), 7.34 (s, 1 H, ArH), 3.38 (s, 1 H, C≡CH), 2.88-2.75 (m, 24 H, $ArC\underline{H}_2$), 1.77-1.62 (m, 24 H, $ArCH_2C\underline{H}_2$), 1.5-1.3 (m, 72 H, CH_2*)*, 1.17 (s, 21 H, TIPS), 0.90 (m, 36 H, CH_3); ^{13}C NMR (126 MHz, CD_2Cl_2): δ = 143.3, 143.1, 142.4, 142.35, and 142.34 ($\underline{C}$Hex), 133.4, 133.2, 132.8 and 132.7 (arom. CH), 123.7, 123.18, 123.17, 123.15, 123.08, and 121.8 ($\underline{C}$C≡C), 106.0 and 95.8 ($\underline{C}$≡$\underline{C}$TIPS), 93.45, 93.41, 93.39, 93.3, and 93.1 (Ar$\underline{C}$≡$\underline{C}$Ar), 82.6 and 81.9 ($\underline{C}$≡$\underline{C}$H), 34.8, 34.55, 34.46, and 34.2 ($Ar\underline{C}H_2$), 32.2, 32.1, 31.3, 31.2, 31.15, 31.08, 30.9, 30.1, 29.8, 29.7, 29.6, 29.5, 23.1, and 23.0 (CH_2), 18.9 ($CH(\underline{C}H_3)_2$), 14.3 ($CH_2\underline{C}H_3$), 11.8 ($\underline{C}H(CH_3)_2$); MALDI-TOF: *m/z* = 1793.27; Anal. Calcd for $C_{131}H_{190}Si$ (1793.047 g/mol): C, 87.75; H, 10.68. Found C, 87.57; H, 10.53.

Alkyne 2_7. See also the general procedure for HOE-removal. *γ*-MnO_2 (822 mg, 9.46 mmol) and powdered KOH (441 mg, 7.86 mmol) were added in six portions to a solution of heptamer **$1b_7$** (400 mg, 0.190 mmol) in Et_2O (50 mL). Column chromatography (4 cm x 20 cm; *n*-pentane/Et_2O, 19:1) of the material, that had been obtained through standard work-up, afforded alkyne **2_7** (352 mg, 90%; R_f = 0.87) as a yellow solid. mp 131-133 °C. ^1H NMR (250 MHz, CD_2Cl_2): δ = 7.41 (m, 10 H, ArH), 7.37 (s, 1 H, ArH), 7.36 (s, 2 H, ArH), 7.34 (s, 1 H, ArH), 3.37 (s, 1 H, C≡CH), 2.90-2.74 (m, 28 H, $ArCH_2$), 1.80-1.60 (m, 28 H, $ArCH_2C\underline{H}_2$), 1.5-1.3 (m, 84 H, CH_2*)*, 1.17

(s, 21 H, TIPS), 0.90 (m, 42 H, CH_3); ^{13}C NMR (63 MHz, CD_2Cl_2): δ = 143.3, 143.1, 142.5, 142.40, and 142.38 (<u>C</u>Hex), 133.4, 133.3, 132.84, and 132.77 (arom. CH), 123.7, 123.3, 123.2, and 121.9 (<u>C</u>C≡C), 106.1 and 95.9 (<u>C</u>≡<u>C</u>TIPS), 93.5, 93.3 and 93.2 (Ar<u>C</u>≡<u>C</u>Ar), 82.7 and 81.9 (C≡CH), 34.8, 34.6, and 34.2 (Ar<u>C</u>H_2), 32.3, 32.1, 31.3, 31.24, 31.16, 31.0, 29.7, 29.5, 23.1, and 23.0 (CH_2), 18.9 (CH(<u>C</u>H_3)$_2$), 14.3 (CH_2<u>C</u>H_3), 11.9 (<u>C</u>H(CH_3)$_2$); MALDI-TOF: *m/z* = 2060.38; Anal. Calcd for $C_{151}H_{218}Si$ (2061.491 g/mol): C, 87.98; H, 10.66. Found C, 87.49; H, 10.70.

Synthesis of alkyne $\mathbf{2_5}$

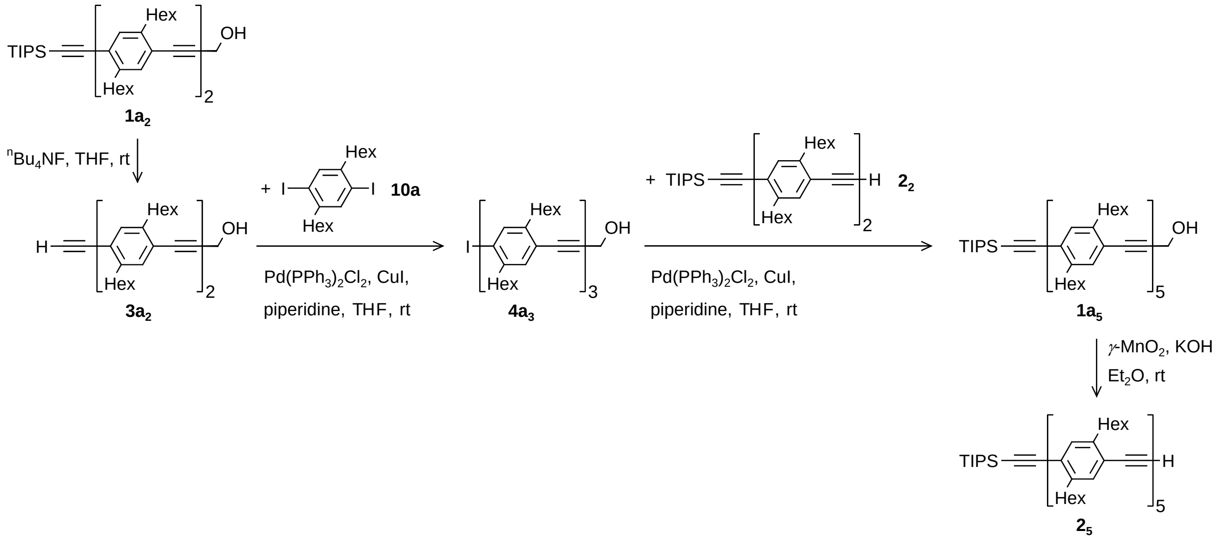

Scheme S3: Synthesis of alkyne $\mathbf{2_5}$.

Alkyne $\mathbf{3a_2}$. To a solution of $\mathbf{1a_2}$ (5.16 g, 6.89 mmol) in THF (250 mL), *n*-Bu_4NF (1 M in THF; 13.8 mL, 13.8 mmol) was added. The reaction mixture was stirred for 2 h at room temperature. Then diethyl ether and water were added, the phases were separated, and the aqueous phase was extracted with diethyl ether. The combined organic phases were washed with water and finally dried (Na_2SO_4). Evaporation of the solvent gave a yellow oil. Column chromatography (5 cm x 20 cm; *n*-

pentane/CH_2Cl_2, 3:1) afforded a yellow oil (4.38 g; R_f = 0.36) containing alkyne **3a₂** and silicon containing reaction products (TIPSOH and/or $TIPS_2O$). ^{1}H NMR (250 MHz, $CDCl_3$): δ = 7.32 and 7.31 (2 s, 3 H, ArH), 7.27 (s, 1 H, ArH), 4.53 (s, 2 H, C$\underline{H}_2$O), 3.29 (s, 1 H, C≡C$\underline{H}$), 2.75 (m, 8 H, ArC$\underline{H}_2$), 1.64 (m, 8 H, ArCH$_2$C$\underline{H}_2$), 1.5-1.3 (m, 24 H, CH_2), 0.87 (m, 12 H, CH_3). The additional singlet at 1.05 ppm with an intensity that corresponds to 15 H is assigned to TIPSOH and/or $TIPS_2O$.

Iodo trimer 4a$_3$. See also the general procedure for alkynyl-aryl coupling. Alkyne **3a$_2$** (4.0 g, ca. 6.7 mmol; The material contained TIPSOH and/or $TIPS_2O$) and diiodobenzene **10a** (33.6 g, 67.4 mmol) were coupled in THF (95 mL) and piperidine (30 mL) mediated by $Pd(PPh_3)_2Cl_2$ (47 mg, 0.07 mmol) and CuI (26 mg, 0.14 mmol). Reaction time: 18 h. Column chromatography (6 cm x 30 cm) gave diiodobenzene **10a** (31.1 g, 93%; eluted with *n*-pentane, R_f = 0.84), iodo trimer **4a$_3$** (3.42 g, 53%; eluted with *n*-pentane/CH_2Cl_2 3:1, R_f = 0.40) as a yellow-brown solid, and a mixture (1.46 g; R_f = 0.07) of dicoupling product **16a$_5$** and oxidative dimerization (Glaser coupling) product of alkyne **3a$_2$**. Analytical data of iodo trimer **4a$_3$**: mp 72-73 °C. ^{1}H NMR (250 MHz, CD_2Cl_2): δ = 7.72 (s, 1 H, ArH ortho to iodo), 7.39 (s, 2 H, ArH), 7.36, 7.34, and 7.31 (3 s, 1 H each, ArH), 4.53 (d, J = 6.1 Hz, 2 H, C$\underline{H}_2$OH), 2.87-2.65 (m, 12 H, ArC$\underline{H}_2$), 1.80-1.58 (m, 12 H, ArCH$_2$C$\underline{H}_2$), 1.37-1.32 (m, 36 H, CH_2), 0.92-0.86 (m, 18 H, CH_3); ^{13}C NMR (63 MHz, CD_2Cl_2): δ = 144.3 ($\underline{C}$Hex ortho to iodo), 143.4, 142.8, 142.48, 142.45, and 142.39 ($\underline{C}$Hex), 139.9 (arom. CH ortho to iodo), 132.82, 132.78, and 132.74 (arom. CH), 123.39, 123.37, 123.2, 123.1, and 122.4 ($\underline{C}$C≡C), 101.2 (C-I), 93.3, 92.9, and 92.8 (Ar$\underline{C}$≡$\underline{C}$Ar), 92.5 and 84.5 ($\underline{C}$≡$\underline{C}$CH$_2$OH), 52.0 (CH$_2$OH), 40.6 (Ar$\underline{C}$H$_2$ ortho to iodo) 34.54, 34.49, 34.3 and 34.2 (Ar$\underline{C}$H$_2$), 32.23, 32.19, 32.11, 32.09, 31.12, 31.08, 30.9, 30.6, 29.7, 29.6, 29.5, 29.4, 23.1 and 23.0 (CH_2), 14.3 (CH_3); Anal. Calcd for $C_{61}H_{87}IO$ (963.271 g/mol): C, 76.06; H, 9.10. Found C, 75.79; H, 9.04.

Pentamer $1a_5$. See also the general procedure for alkynyl-aryl coupling. Iodo trimer $\mathbf{4a_3}$ (1.186 g, 1.23 mmol) and alkyne $\mathbf{2_2}$ (844 mg, 1.17 mmol; Please note: The alkyne $\mathbf{2_2}$ must have been used in excess. A miscalculation made us using an insufficient amount of this alkyne with the consequences described below.) were coupled in THF (40 mL) and piperidine (12 mL) with $Pd(PPh_3)_2Cl_2$ (9.2 mg, 0.013 mmol) and CuI (5.3 mg, 0.028 mmol) as the catalysts. Reaction time: 18.5 h. The crude product was dissolved in CH_2Cl_2 (3 mL). Upon addition of MeOH a yellow precipitate (1.8 g) formed which was isolated and dried in vacuo. Column chromatography (3 cm x 4 cm; Et_2O/*n*-pentane 1:9) gave a mixture (1.72 g) of the pentamer $\mathbf{1a_5}$ along with the oxidative dimerization (Glaser coupling) product of alkyne $\mathbf{2_2}$ and iodo trimer $\mathbf{4a_3}$. A second fraction (25 mg) contained the iodo trimer $\mathbf{4a_3}$ along with some unidentified compounds. Because pentamer $\mathbf{1a_5}$ and iodo trimer $\mathbf{4a_3}$ are practically inseparable, a second alkynyl-aryl coupling was performed, with the material of the first chromatography fraction and alkyne $\mathbf{2_2}$ (84 mg, 0.12 mmol) in THF (40 mL) and piperidine (12 mL), with $Pd(PPh_3)_2Cl_2$ (2.3 mg, 0.003 mmol) and CuI (2.3 mg, 0.012 mmol) as the catalysts, and a reaction time of 18 h. The crude product was adsorbed onto a small quantity of silica gel by dissolving it in CH_2Cl_2, adding silica gel to this solution, and removing the solvent at reduced pressure. The free flowing powder was transferred onto the top of a silica gel column (3 cm x 28 cm). Elution with Et_2O/*n*-pentane 1:9 afforded a mixture (151 mg; R_f = 0.98-0.83) consisting mainly of oxidative dimerization (Glaser coupling) product of alkyne $\mathbf{2_2}$ and residual alkyne $\mathbf{2_2}$. After changing the solvent to Et_2O/*n*-pentane 3:7, a mixture (8 mg) of pentamer $\mathbf{1a_5}$ (R_f = 0.38) and a 1,1-disubstituted ethene (R_f = 0.48) that had resulted from the addition of the C_{ethyne}-H moiety of alkyne $\mathbf{2_2}$ onto the monosubstituted triple bond of alkyne $\mathbf{2_2}$ was eluted. Finally, pentamer $\mathbf{1a_5}$ (1.54 g, 81%; R_f = 0.38), a yellow solid, was obtained. Analytical data of pentamer $\mathbf{1a_5}$: mp 115-117°C. ^{1}H NMR (250 MHz,

CD_2Cl_2): δ = 7.403, 7.399, and 7.392 (3 s, 6 H, ArH), 7.36 (s, 1 H, ArH), 7.35 (s, 1 H, ArH), 7.33 (s, 1 H, ArH), 7.30 (s, 1 H, ArH), 4.52 (d, J = 5.9 Hz, 2 H, $C\underline{H}_2OH$), 2.89-2.71 (m, 20 H, $ArC\underline{H}_2$), 1.79-1.60 (m, 20 H, $ArCH_2C\underline{H}_2$), 1.5-1.3 (m, 60 H, CH_2), 1.17 (s, 21 H, TIPS), 0.90 (m, 30 H, CH_3); ^{13}C NMR (63 MHz, CD_2Cl_2): δ = 143.2, 142.8, 142.5, 142.41 and 142.39 ($\underline{C}$Hex), 133.3, 132.9, 132.85 and 132.79 (arom. CH), 123.4, 123.28, 123.25 and 122.4 ($\underline{C}C{\equiv}C$), 106.1 and 95.9 ($\underline{C}{\equiv}\underline{C}$TIPS), 93.51, 93.47, 93.35, and 93.31 ($Ar\underline{C}{\equiv}\underline{C}Ar$), 92.5 and 84.5 ($\underline{C}{\equiv}\underline{C}CH_2OH$), 52.0 ($CH_2OH$), 34.8, 34.6 and 34.3 ($Ar\underline{C}H_2$), 32.3, 32.1, 31.4, 31.3, 31.2, 31.1, 31.0, 29.78, 29.72, 29.67, 29.5, 23.11, and 23.05 (CH_2), 18.9 ($CH(\underline{C}H_3)_2$), 14.3 ($CH_2\underline{C}H_3$), 11.9 ($\underline{C}H(CH_3)_2$); Anal. Calcd for $C_{112}H_{164}OSi$ (1554.629 g/mol): C, 86.53; H, 10.63. Found C, 86.50; H, 10.65.

Alkyne 2_5. See also the general procedure for HOM-removal. γ-MnO_2 (3.69 g, 42.5 mmol) and powdered KOH (1.19 g, 21 mmol) were added in four portions to a solution of pentamer **$1a_5$** (971 mg, 0.62 mmol) in Et_2O (50 mL). Column chromatography (4 cm x 20 cm; *n*-pentane/Et_2O, 3:1) of the material that had been obtained through standard work-up afforded alkyne **2_5** as a yellow solid (694 mg, 73%; R_f = 0.96). mp 91-93 °C; ^1H NMR (500 MHz, CD_2Cl_2): δ = 7.40-7.39 (3 s, 6 H, ArH), 7.36 (s, 1 H, ArH), 7.35 (s, 2 H, ArH), 7.33 (s, 1 H, ArH), 3.37 (s, 1 H, $C{\equiv}CH$), 2.87-2.74 (m, 20 H, $ArC\underline{H}_2$), 1.75-1.61 (m, 20 H, $ArCH_2C\underline{H}_2$), 1.5-1.3 (m, 60 H, CH_2), 1.16 (s, 21 H, TIPS), 0.89 (m, 30 H, CH_3); ^{13}C NMR (126 MHz, CD_2Cl_2): δ = 143.3, 143.1, 142.4, 142.33, and 142.31 ($\underline{C}$Hex), 133.3, 133.2, 132.8, and 132.7 (arom. CH), 123.6, 123.18, 123.16, 123.14, 123.0, and 121.7 ($\underline{C}C{\equiv}C$), 106.0 and 95.8 ($\underline{C}{\equiv}\underline{C}$TIPS), 93.39, 93.36, 93.34, 93.2 and 93.1 ($Ar\underline{C}{\equiv}\underline{C}Ar$), 82.6 and 81.9 ($\underline{C}{\equiv}\underline{C}H$), 34.7, 34.5, 34.4, and 34.2 ($Ar\underline{C}H_2$), 32.2, 32.0, 31.3, 31.2, 31.1, 31.0, 30.9, 29.7, 29.6, 29.5, 23.04, and 22.98 (CH_2), 18.8 ($CH(\underline{C}H_3)_2$), 14.2 ($CH_2\underline{C}H_3$), 11.7

($\underline{C}H(CH_3)_2$); MALDI-TOF: *m/z* = 1523.77; Anal. Calcd for $C_{111}H_{162}Si$ (1524.603 g/mol): C, 87.45; H, 10.71. Found C, 87.59; H, 10.71.

Synthesis of polar tagged building block 10b

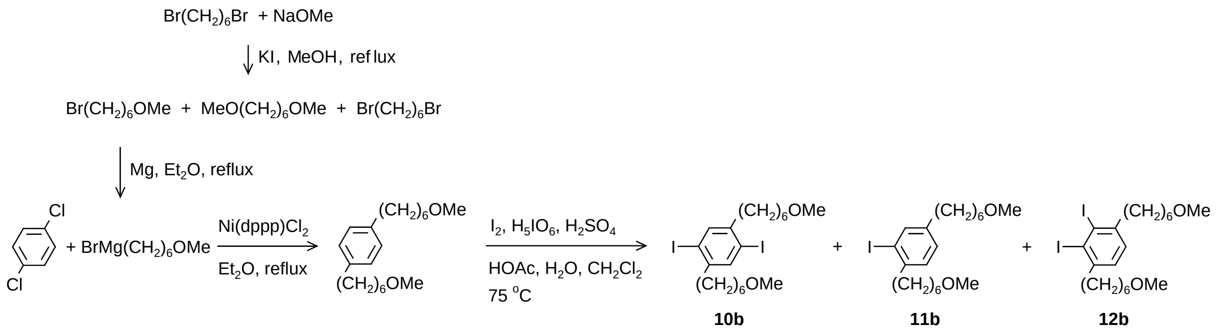

Scheme S4: Synthesis of polar tagged building block **10b**.

6-Bromohexylmethylether. The procedure was based on that described by Rau and Rehahn [7]. In contrast to Rau and Rehahn, we were not able to separate the three compounds in the crude product by distillation. Therefore, we switched to column chromatography.

Sodium methanolate (57.3 g, 1.06 mol) was suspended in methanol (300 mL) with ice cooling. This suspension was added dropwise to a mixture of 1,6-dibromohexane (156 mL, 1.03 mol) and KI (1.0 g, 6 mmol) in diethyl ether (100 mL) with ice cooling. The reaction mixture was refluxed for 45 h. After cooling to room temperature the reaction mixture was poured into water (300 mL). The aqueous phase was extracted with diethyl ether, the combined organic phases were washed several times with water, dried ($MgSO_4$), and the solvent was removed. The crude product was split into two portions for column chromatography (7 cm x 25 cm). Elution with *n*-pentane gave 1,6-dibromohexane (25 g, 10%) as the first fraction. After changing the solvent (*n*-pentane/CH_2Cl_2 1:1) a mixture of 1,6-dibromohexane and 1-bromo-6-methoxyhexane

(37 g) and finally pure 1-bromo-6-methoxyhexane (70 g, 35%) were obtained as colorless liquids. 1,6-Dimethoxyhexane was left on the column. The compounds can be made visible on TLC by staining with ammonium 8-anilinonaphthalene-1-sulfonate [8]. ^{1}H NMR (250 MHz, $CDCl_3$): *δ* = 3.39 (t, *J* = 6.8 Hz, 2 H, CH_2Br), 3.35 (t, *J* = 6.5 Hz, 2 H, OCH_2), 3.31 (s, 3 H, OCH_3), 1.85 and 1.57 (2 m, 2 H each, CH_2), 1.40 (m, 4 H, CH_2); ^{13}C NMR (63 MHz, $CDCl_3$): *δ* = 72.6 ($\underline{C}H_2O$), 58.5 ($O\underline{C}H_3$), 33.8, 32.7, 29.4, 28.0, and 25.3 (CH_2); Anal. Calcd for $C_7H_{15}BrO$ (195.100): C 43.09; H 7.75. Found: C 43.14; H 7.65.

1,4-Bis(6-methoxyhexyl)benzene. Starting from 1-bromo-6-methoxyhexane (37.5 g, 192 mmol) and 1,4-dichlorobenzene (11.3 g, 77 mmol) and following the procedure given by Rau and Rehahn [7], 1,4-bis(6-methoxyhexyl)benzene (13 g, 57%) was obtained as a colorless liquid. ^{1}H NMR (250 MHz, $CDCl_3$): *δ* = 7.07 (s, 4 H, ArH), 3.36 (t, *J* = 6.5 Hz, 4 H, OCH_2), 3.32 (s, 6 H, OCH_3), 2.57 (t-shaped signal, *J* = 7.7 Hz, 4 H, $ArC\underline{H}_2$), 1.36 and 1.58 (2 m, 16 H, CH_2); ^{13}C NMR (63 MHz, $CDCl_3$): *δ* = 139.9 ($\underline{C}_{Ar}Alk$), 128.2 (arom. CH), 72.8 (OCH_2), 58.5 (OCH_3), 35.4, 31.4, 29.5, 29.1, and 26.0 (CH_2).

1,4-Diiodo-2,5-bis(6-methoxyhexyl)benzene (10b). To a solution of 1,4-bis(6-methoxyhexyl)benzene (11.66 g, 38.0 mmol) in acetic acid (100 mL) were added water (18 mL), conc. H_2SO_4 (3.5 mL), CH_2Cl_2 (15 mL), I_2 (8.05 g, 31.7 mmol), and H_5IO_6 (3.56 g, 15.6 mmol). The reaction mixture was stirred for 17 h at 75 °C. After cooling to room temperature, diethyl ether, *n*-pentane and aqueous 10% Na_2SO_3 were added to the reaction mixture. The phases were separated and the aqueous phase was extracted with a mixture of diethyl ether and *n*-pentane. The combined organic phases were washed with water, dried ($MgSO_4$) and the solvents were removed. The residue contained diiodination product **10b**, monoiodination product

11b [9], and the constitutional isomeric diiodination product **12b** [9] in a ratio of about 16:1:1. It was adsorbed onto a small quantity of silica gel by dissolving it in CH_2Cl_2, adding silica gel to this solution, and removing the solvent at reduced pressure. The free flowing powder was transferred onto the top of a silica gel column (5.5 cm x 42 cm). Elution (*n*-pentane/Et_2O 5:1) followed by threefold recrystallization from methanol (40 mL each time) provided 1,4-diiodo-2,5-bis(6-methoxyhexyl)benzene (**10b**) (13.4 g, 63 %) as colorless needles. mp 53 ^{o}C; ^{1}H NMR (250 MHz, $CDCl_3$): δ = 7.58 (s, 2 H, C_6H_4), 3.37 (t, J = 6.5 Hz, 4 H, OCH_2), 3.32 (s, 6 H, OCH_3), 2.59 (t-shaped signal, J = 7.7 Hz, 4 H, $ArC\underline{H}_2$), 1.56 and 1.38 (2 m, 8 H each, CH_2); ^{13}C NMR (63 MHz, $CDCl_3$): δ = 144.7 ($\underline{C}_{Ar}$Alkyl), 139.3 (arom. CH), 100.3 (C-I), 72.8 (OCH_2), 58.5 (OCH_3), 39.7, 30.1, 29.5, 29.1, and 25.9 (CH_2); Anal. Calcd for $C_{20}H_{32}I_2O_2$ (558.284): C 43.03; H 5.78. Found C 42.91; H 5.70.

Synthesis of 14b

See also the general procedure for alkynyl-aryl coupling. Diiodobenzene **10b** (98 mg, 0.18 mmol) and alkyne **13** (372 mg, 0.37 mmol) were coupled in THF (5 mL) and piperidine (1.5 mL) with $Pd(PPh_3)_2Cl_2$ (3.8 mg, 0.005 mmol) and CuI (3.3 mg, 0.017 mmol) as the catalysts. Reaction time: 19 h. Column chromatography (the crude product was dissolved in CH_2Cl_2/*n*-pentane 1:1; 3.5 cm x 24 cm; CH_2Cl_2/*n*-pentane 1:3) afforded a first fraction (2 mg; R_f = 0.77). Subsequent elution with CH_2Cl_2/*n*-pentane 1:3 and finally with CH_2Cl_2/*n*-pentane 6:4 gave a mixture of coupling product **14b** and the Glaser coupling product **15**. These two components were separated by chromatography (the yellow solid containing **14b** and **15** was dissolved in CH_2Cl_2/*n*-pentane 6:4; 3 cm x 35 cm). Elution first with CH_2Cl_2/*n*-pentane 1:4 then with CH_2Cl_2/*n*-pentane 1:2 gave a fraction (14 mg; R_f(CH_2Cl_2/*n*-pentane 6:4) = 0.71) consisting essentially of the Glaser coupling product **15** [5]. Faint additional spots on

the TLC (R_f(CH_2Cl_2/*n*-pentane 6:4) = 0.62, 0.23) and a very careful look at the ^{1}H NMR spectra of this fraction revealed that some of the phenol groups had been deprotected during chromatography. Elution with CH_2Cl_2/*n*-pentane 1:1 afforded a mixture (2 mg, R_f(CH_2Cl_2/*n*-pentane 6:4) = 0.50 (very faint spot), 0.38 (very faint spot), and 0.23 (intense spot)) of compounds, mainly **15** and deprotected **15**. Upon changing the ratio of CH_2Cl_2 and *n*-pentane to 6:4, **14b** (259 mg, 64%; R_f(CH_2Cl_2/*n*-pentane 6:4) = 0.29), a yellow solid, was obtained. A very faint spot on TLC with R_f(CH_2Cl_2/*n*-pentane 6:4) = 0.04 indicated that this fraction was contaminated by traces of (partially) deprotected **14b**. A last fraction (55 mg; R_f(CH_2Cl_2/*n*-pentane 6:4) = 0.29, 0.04) consisting of **14b** and (partially) deprotected **14b** (ratio of ArOTHP and ArOH was 1.0:1.4; determined by ^{1}H NMR spectroscopy) was eluted after changing the solvent to pure CH_2Cl_2 and finally to Et_2O. Analytical data of **14b**: mp 155-156°C; ^{1}H NMR (500 MHz, $CDCl_3$): δ = 7.45 (half of AA'XX' spin system, 4 H, H meta to OTHP), 7.37 (s, 10 H, C_6H_2), 7.35 (s, 4 H, C_6H_2), 7.04 (half of AA'XX', 4 H, H ortho to OTHP), 5.46 (t-shaped signal, J = 2.9 Hz, 2 H, O_2CH), 3.89 and 3.62 (2 m, 2 H each, OCH_2 of THP), 3.35 (t, J = 6.7 Hz, 4 H, $C\underline{H}_2OMe$), 3.31 (s, 6 H, OCH_3), 2.83-2.78 (m, 28 H, $ArC\underline{H}_2$), 2.00 (m, 2 H, CH_2), 1.87 (m, 4 H, CH_2), 1.71 (m, 32 H, CH_2), 1.59 (m, 6 H, CH_2), 1.42 (m, 30 H, CH_2), 1.33 (m, 50 H, CH_2), 0.88 (m, 36 H, $C\underline{H}_3$); ^{13}C NMR (126 MHz, $CDCl_3$): δ = 157.1 ($\underline{C}OTHP$), 142.1, 141.94, 141.90, 141.85, and 141.78 ($\underline{C}_{Ar}Alk$), 132.8, 132.4, and 132.1 (CH of C_6H_2 and CH meta to OTHP), 122.89, 122.86, 122.83, 122.80, 122.71, 122.68, and 122.4 ($\underline{C}$C≡C of C_6H_2), 116.45 (CH ortho to OTHP), 116.43 ($\underline{C}$C≡C of C_6H_4), 96.2 (O_2CH), 94.1, 93.16, 93.13, 93.11, 93.01, 92.99, 92.8, and 87.2 (C≡C), 72.8 ($\underline{C}H_2OMe$), 62.0 (OCH_2 of OTHP), 58.5 (OCH_3), 34.2, 34.1, 31.83, 31.77, 30.7, 30.6, 30.2, 29.6, 29.4, 29.3, 26.1, 25.1, 22.7, and 18.6 (CH_2), 14.1 ($CH_2\underline{C}H_3$); Anal. Calcd for $C_{166}H_{226}O_6$ (2317.628 g/mol): C, 86.03; H, 9.83. Found C, 86.14; H, 9.66.

References

[1] Thulin, B.; Wennerström, O. *Acta Chem. Scand., Ser. B* **1983,** *37*, 589–595. doi: 10.3891/acta.chem.scand.37b-0589

[2] Own data.

[3] Kukula, H.; Veit, S.; Godt. A. *Eur. J. Org. Chem.* **1999,** 277–286. doi: 10.1002/(SICI)1099-0690(199901)1999:1<277::AID-EJOC277>3.0.CO;2-R

[4] Rehahn, M.; Schlüter, A.-D.; Feast, W. J. *Synthesis* **1988,** 386–388.

[5] Godt, A.; Schulte, M.; Zimmermann, H.; Jeschke, G. *Angew. Chem.* **2006,** *118*, 7722–7726. doi: 10.1002/ange.200602807

[6] Fatiadi, A. J. *Synthesis* **1976,** 65–104. doi: 10.1055/s-1976-23961

[7] Rau, I. U.; Rehahn M. *Acta Polymer.* **1994,** *45*, 3–13. doi: 10.1002/actp.1994.010450103

[8] Jork, H.; Funk, W.; Fischer, W.; Wimmer, H. *Dünnschicht-Chromatographie*, Vol. 1, VCH, Germany, 1989; p 191.

[9] The byproducts **11b** and **12b** were identified through the ^{1}H NMR signals assigned to the aromatic and benzylic protons. Shape and position of these signals are the same as of those from **11a** and **12a**, respectively (see Results and Discussion).
